# GASTROESOPHAGEAL SYMPTOMS AFTER LAPAROSCOPIC GASTRIC BYPASS: MISTAKES IN PERFORMING THE PROCEDURE?

**DOI:** 10.1590/0102-672020210002e1657

**Published:** 2022-06-17

**Authors:** Italo BRAGHETTO, Owen KORN, Luis GUTIÉRREZ, Andrés TORREALBA, Jorge ROJAS

**Affiliations:** 1 Department of Surgery, Hospital “Dr. José J Aguirre”, Faculty of Medicine, University of Chile, Santos Dumont 999, Santiago, Chile

**Keywords:** Gastric bypass, Anatomy, Gastroesophageal reflux, Signs and Symptoms, Derivação gástrica, Anatomia, Refluxo gastroesofágico, Sinais e sintomas

## Abstract

Laparoscopic Roux-en-Y gastric bypass (LGB) is the recommended procedure for morbidly obese patients with gastroesophageal reflux disease (GERD). However, there have been reported gastroesophageal reflux symptoms or esophagitis after LGB. Few functional esophageal studies have been reported to date.

**AIM:**

To evaluate the anatomic and physiologic factors contributing to the appearance of these problems in patients who underwent LGB.

**METHODS:**

This prospective study included 38 patients with postoperative gastroesophageal reflux symptoms submitted to LGB. They were subjected to clinical, endoscopic, radiologic, manometric, and 24-h pH-monitoring evaluations.

**RESULTS:**

Eighteen (47.4%) of 38 patients presented with heartburn or regurgitation, 7 presented with pain, and 4 presented with dysphagia. Erosive esophagitis was observed in 11 (28.9%) patients, and Barrett’s esophagus (5.7%) and jejunitis (10.5%) were also observed. Hiatal hernia was the most frequent finding observed in 15 (39.5%) patients, and most (10.5%) of these patients appeared with concomitant anastomotic strictures. A long blind jejunal loop was detected in one (2.6%) patient. Nearly 75% of the patients had hypotensive lower esophageal sphincter (9.61±4.05 mmHg), 17.4% had hypomotility of the esophageal body, and 64.7% had pathologic acid reflux (% time pH <4=6.98±5.5; DeMeester’s score=32.4±21.15).

**CONCLUSION:**

Although rare, it is possible to observe gastroesophageal reflux and other important postoperative symptoms after LGB, which are associated with anatomic and physiologic abnormalities at the esophagogastric junction and proximal gastric pouch.

## RESUMO - RACIONAL

O *bypass* gástrico laparoscópico em Y de Roux (BGL) é o procedimento de escolha para pacientes com obesidade mórbida com doença do refluxo gastroesofágico (DRGE). No entanto, foram relatados sintomas de refluxo gastroesofágico ou esofagite após BGL. Poucos estudos esofágicos funcionais foram relatados até o momento.

**OBJETIVO:**

Avaliar os fatores anatômicos e fisiológicos que contribuem para o surgimento desses problemas em pacientes submetidos a BGL.

**MÉTODOS:**

Este estudo prospectivo incluiu 38 pacientes submetidos a BGL apresentando sintomas de refluxo gastroesofágico pós-operatório. Eles foram submetidos a avaliações clínicas, endoscópicas, radiológicas, manométricas e de pHmetria de 24 horas.

**RESULTADOS:**

18/38 (47,4%) dos pacientes apresentavam azia ou regurgitação, 7 apresentavam dor e 4 pacientes apresentavam disfagia. Esofagite erosiva observada em 11 pacientes (28,9%), esôfago de Barrett (5,7%) e jejunite (10,5%) também foram observadas. A hérnia de hiato foi o achado mais frequente observado em 15 pacientes (39,5%), a maioria deles com estenoses anastomóticas concomitantes (10,5%). Alça jejunal cega longa foi detectada em 1 paciente (2,6%). Quase 75% dos pacientes apresentavam esfíncter esofágico inferior hipotensivo (9,61- + 4,05 mmHg), 17,4% tinham hipomotilidade do corpo esofágico e 64,7% apresentavam refluxo ácido patológico (% tempo pH <4 = 6,98- + 5,5; pontuação de DeMeester = 32,4- + 21,15).

**CONCLUSÃO:**

Embora raros, é possível observar refluxo gastroesofágico e outros sintomas pós-operatórios importantes após BGL, os quais estão associados a alterações anatômicas e fisiológicas na junção esofagogástrica e bolsa gástrica proximal.

## INTRODUCTION

As laparoscopic sleeve gastrectomy is contraindicated in morbidly obese patients with gastroesophageal reflux disease (GERD), laparoscopic Roux-en-Y gastric bypass (LGB) is the preferred procedure for these patients ^5^ ^,^ ^7^ ^,^ ^33^ ^,^ ^35^ ^,^ ^36^ ^,^ ^39^ ^,^ ^42^. Reported literature suggest LGB can also be performed in patients with gastroesophageal reflux symptoms (GERD) who had undergone sleeve gastrectomy ^1^ ^,^ ^2^ ^,^ ^11^ ^,^ ^15^ ^,^ ^24^.

However, some publications have reported the appearance or persistence of gastroesophageal reflux symptoms and erosive esophagitis (EE) after LGB ^1^ ^,^ ^2^ ^,^ ^5^ ^,^ ^7^ ^,^ ^8^ ^,^ ^15^ ^,^ ^22^ ^,^ ^24^ ^,^ ^19^ ^,^ ^45^. These articles mainly focused on the symptoms or endoscopic findings. Objective evidence based on anatomic and physiologic studies is scarce. 

This study aimed to evaluate the anatomic and physiologic abnormalities contributing to the appearance of hiatal hernia (HH) and reflux disease after LGB.

## METHODS

This prospective study included 38 patients with reflux symptoms after LGB for morbid obesity; 27 of them have undergone primary LGB and 11 after conversion to LGP due to intractable reflux symptoms caused by sleeve gastrectomy. Despite the procedure and medical treatment with proton-pump inhibitors (PPIs), these patients continue to present symptoms after the operation. Thirty-three patients were females and two were males, with a mean age of 43.9 years (range 27-72 years. Body mass index (BMI) before LGB was 45.2+4.5 kg/m^2^, they showed satisfactory weight loss after surgery, and the BMI decreased to 27.8+4.7 kg/m^2^ after the operation. Twenty-three patients were initially operated on by us [comprised of 500 gastric bypasses performed by our team (0.05%)]. The other patients were operated on by surgeons from other surgical units. Due to the symptoms, they consulted us directly or were referred to us for evaluation or treatment; therefore, we do not know the total universe in those hospitals.

**Figure 1 -:**
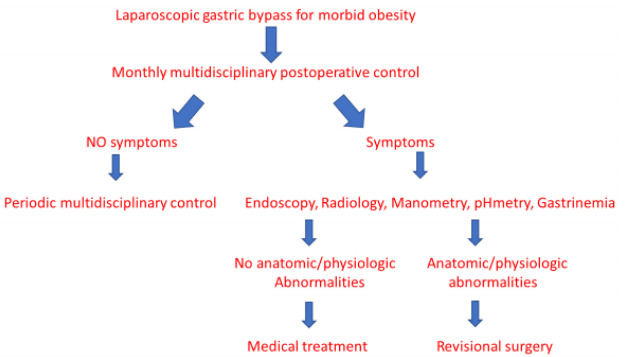
Algorithm for postoperative evolution routinely performed in patients who had undergone laparoscopic gastric bypass.

The patients were examined using endoscopy, radiology, manometry, and 24-h pH-monitoring evaluation. Some patients were also subjected to serum gastrin measurements (Figure 1). This evaluation was performed between 6 and 12 months after LGB, which is usually followed when the patients start developing postoperative symptoms.

In the multidisciplinary postoperative control, patients were submitted to the following assessment:

- **Clinical questionnaire:** In this procedure, heartburn symptoms, regurgitation, retrosternal pain, dysphagia, vomiting, or respiratory symptoms were recorded.
- **Endoscopy:** This procedure was performed using a CV 190 Olympus flexible gastroscope after a 12-h fasting and pharyngeal anesthesia with lidocaine. An examination was performed to visualize the squamous-columnar junctions and establish the presence of erosive esophagitis as defined by the Los Angeles classification. The presence and size of HH were recorded. Endoscopic Barrett’s esophagus (BE) was confirmed by histology ^16^. The presence of patulous cardia was also recorded.
- **Radiologic evaluation:**Patients were subjected to a barium swallow and computed tomography (CT) scan to evaluate the anatomic aspect of the cardia and the presence of HH and its size (measured in cm). The size of the vertebral body (3 cm) and evidence of radiological reflux were used as references. The capacity of the gastric pouch (measured in cc) was calculated during the acquisition of images of the vertical length of the pouch, considering the frontal and lateral diameters of the contrasted gastric area. The characteristics of gastrojejunal anastomosis and radiological gastric emptying were also recorded. It is important to emphasize that there was no history of surgery in the hiatus during the initial operation.
- **Manometric and 24-h pH-monitoring evaluation:** In only 17 patients, it was possible to evaluate the manometric characteristic of the esophageal body and LES: Standard or high-resolution manometry was performed after 12-h fasting and before pH monitoring. The resting pressure, abdominal length, the total length of the lower esophageal sphincter (LES), and the amplitude of the esophageal contractile waves were measured. Hypotensive LES was defined as a resting pressure of <13 mmHg, and an incompetent LES was <2 cm. Hypomotility or ineffective peristalsis was defined as esophageal waves with amplitudes of <30 mmHg. The characteristic of esophageal peristalsis was evaluated after 10 wet swallows ^16^. Acid-reflux evaluation was performed after 12-h fasting and discontinued the PPI treatment 8 days before the study. A catheter was introduced through the nose into the stomach. The tip was placed 5 cm proximal to the upper border of the LES ^30^.
- **Serum gastrin:** It was measured in the gastrointestinal laboratory unit using an Immulite 2000 Gastrin Siemens Medical Solutions Diagnostics kit (Siemens Healthineers, Erlangen, Germany). PPI treatment was discontinued at least 8 days before, and blood samples were collected after 8-h fasting. Normal gastrin level was considered <100 pg/mL ^25^.

All patients signed the informed written consent.

Inclusion criteria were:

1. patients with severe symptoms who had previously undergone LGB,
2. patients who did not respond to PPIs treatment after LGB,
3. patients with complete objective evaluations, and
4. patients with no history of hiatus surgery at initial surgery.

Exclusion criteria were:

1. no symptoms, esophagitis, or HH after LGB,
2. patients who underwent other upper esophagogastric surgery, and
3. other postoperative complications after LGB.

## RESULTS

The clinical presentation and endoscopic findings are presented in Table 1. Reflux symptoms associated with HH and erosive esophagitis were seen in most patients. Dysphagia was observed in patients presenting with HH and anastomotic stricture and pain associated with jejunitis and Barrett’s ulcer. Vomiting was observed in patients with HH, anastomotic stricture; one patient presented obstruction of the alimentary jejunal loop because of lateral compression secondary to a much dilated long blind jejunal loop.

**Table 1 -:** Gastroesophageal symptoms after laparoscopic gastric bypass and endoscopic findings (n=38).

Heartburn/regurgitation	18 (47.4%)	Normal endoscopy (n=8) Esophagitis (n=9) Esophagitis and Barrett’s esophagus (n=1) Hiatal hernia (n=9)* Patulous cardia (n=3)*
Pain	7 (18.4%)	Barrett’s esophagus and an ulcer (n=1) Jejunitis (n= 4) Hiatal hernia (n=1) Normal endoscopy (n=1) (motility disorder)
Dysphagia	4 (10.5%)	Hiatal hernia (n=3) Normal endoscopy (n=1) (motility disorder)
Vomiting*	7 (26.3%)	Hiatal hernia (n=2) Anastomotic stricture (n=4) Long blind jejunal loop (n=1)
Nausea	1 (2.6%)	Normal endoscopy (n=1)

*Concomitant findings.

The endoscopic findings are presented in Table 2. Normal endoscopy was observed in 11 (28.9%) patients. The most frequent findings were the presence of HH (39.5%) and EE (28.9%), some of them in combination with HH, patulous cardia, and Barrett´s esophagus. Jejunitis and anastomotic strictures were also observed.

**Table 2 -:** Endoscopic findings in 40 patients presenting gastroesophageal symptoms after laparoscopic gastric bypass.

Endoscopic findings	n (%)
Normal	7 (17.5)
Abnormal	33 (82.5)
Hiatal hernia	16 (40)
Patulous cardia	4
Esophagitis*	12(30)
Grade A	8
Grade B	2
Grade C	1
Grade D	1
Concomitant Barrett´s esophagus*	3* (1 with esophageal ulcer)
Jejunitis*	5*
Anastomotic stricture*	4* (1 lateralized anastomosis with stricture)
Long blind jejunal loop	2

*Concomitant findings.

**Figure 2 -:**
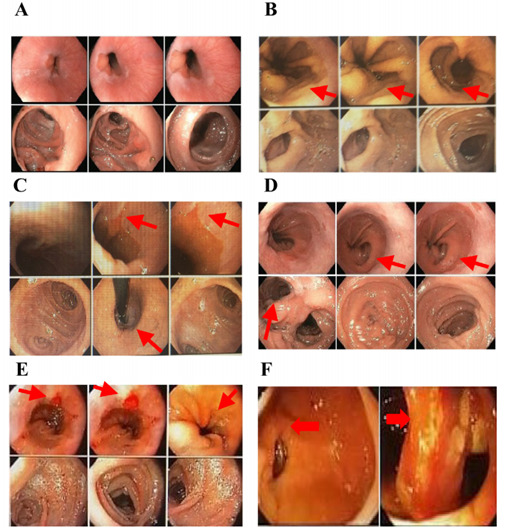
Endoscopic findings after laparoscopic gastric bypass. (A) Normal findings after laparoscopic gastric bypass, (B) hiatal hernia post-LGB (arrows), (C) esophagitis and patulous cardia (arrows), (D) hiatal hernia, the island of columnar epithelium, and long blind jejunal loop (arrows), (E) erosive esophagitis and Barrett´s esophagus (arrows), and (F) lateralized gastrojejunostomy with stricture (arrows).

Figure 2 shows several images of endoscopic control after LGB, comparing the normal endoscopy versus different abnormal anatomic defects after LGB.

Generally, anatomic abnormalities after surgery are associated with pathophysiological changes. Hypotensive LES was observed in 13 (74.6%) patients. Lower esophageal sphincter resting pressure (9.61+4.05 mmHg) was observed in 17.4% of the cases associated with ineffective motility and had an esophageal wave amplitude of 62.3±5.63 mmHg. Normal peristalsis was observed in 88.2% of the patients (8/10 wet swallows generate peristaltic waves). Pathologic acid reflux was observed in 64.7% of the patients (%time pH <4=6.98±5.5, DeMeester’s score=32.4±21.15) (Table 3). These findings might explain the development of reflux symptoms and esophagitis.

**Table 3 -:** Manometry and 24-h pH findings in 17 patients presenting gastroesophageal symptoms after laparoscopic gastric bypass.

Manometry	
Number of patients with normal LOSP	4 (23.5%)
Number of patients with incompetent LOS	13 (76.4%)
LOSP (mmHg)	Mean 9.61±4.05 (range 2.5-16)
LOS (cm)	Mean 3.10±0.6 (range 2.0-4.0)
Hypomotility of esophageal body	3 (17.4%)
Amplitute proximal esophageal waves	Mean 62.3±5.63
Normal peristalsis	88.2% (9/10 peristaltic wave)*
**24-HR pH monitoring**	
Number of patients with normal pH monitoring	6 (35.2%)
Number of patients with pathologic acid refluxo	11 (64.7%)
% time pH <4	Mean 6.98±5.5 (range 0.6-20.2)
DeMeester’s score	Mean 32.4±21.15 (range 9.9-64)

*After wet swallow.

**Figure 3 -:**
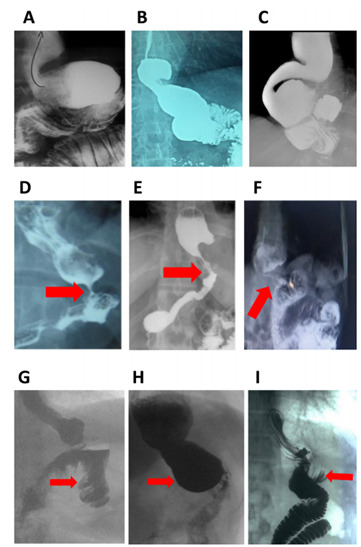
Radiological images of hiatal hernia after laparoscopic gastric bypass detected in our patients. (A) Large gastric pouch and reflux, (B) hiatal hernia and large gastric pouch, (C) hiatal hernia with intrathoracic gastric pouch and gastrojejunal anastomosis, (D) large gastric pouch and hiatal hernia with anastomotic stricture, (E) hiatal hernia and anastomotic stricture, (F) hiatal hernia and anastomotic stricture. (arrows), and (G, H, I) Candy cane images.

The radiological study confirmed the presence of HH in 15 patients, which were found during the endoscopic study (HH was >5 cm in five of them), one of them with complete intramediastinal gastric pouch and gastrojejunal anastomosis. An enlarged gastric pouch with augmented gastric capacity >200 cc was observed in nine patients (range 215-680 cc). No Roux limb length <150 cm was detected, as measured by CT assessment (Table 4; Figure 3)

**Table 4 -:** Radiological findings in 38 patients presenting gastroesophageal symptoms after laparoscopic gastric bypass.

	n
Normal findings	18 (47.4%)
Hiatal hérnia	15 (39.5%)
Size	
>5 cm	5
<5 cm	10
Anastomotic stricture	4 (10.5%)
Candy cane image	1 (2.6%)
Proximal Gastric stump size*	
>5 cm	9 (23.7%)
<5 cm	29 (76.3%)
Gastric capacity (mean 228±133 cc)	
<200 cc	9 (range 215-680)
>200 cc	29 (range 92-195)

*Vertical measurement.

**Volume measured during images acquisition,

Serum gastrin levels were normal in 16 patients (164±195.9 pg/mL, range 23-105 pg/mL), and an increased serum gastrin level was detected in only one patient (509 pg/mL).

Twenty-nine patients were treated with dietary modification and restriction of irritable spiced foods and PPIs (40 mg/day).

**Figure 4 -:**
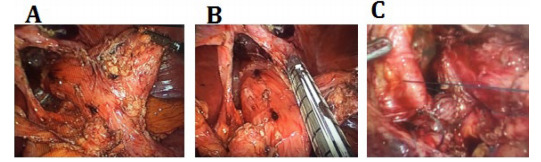
Laparoscopic visualization of hiatal hernia after LGB.

Revisional surgery was performed on nine patients with HH repair and gastric re-resection (one of them with a long blind jejunal loop). Figure 4 shows the resection of the herniated portion of the gastric pouch. All patients reported satisfaction and absence of symptoms at least 1 year after surgery.

(A) Redundant herniated gastric pouch taken down from the intramediastinal position, (B) resection of the herniated redundant pouch, and (C) closure of diaphragmatic crura behind the esophagus.

## DISCUSSION

The prevalence of EE after sleeve gastrectomy was as high as 39% compared with 19% after LGB, and severe esophagitis was 10.7% versus 3.1% after LGB; SG continued to be associated with higher odds of EE compared with LGB (OR=2.47, p=0.001). In addition, conversion to LGB after LSG due to GERD is clinically relevant and may be a feasible solution if patients have ongoing GERD refractory to medical therapy. Ninety-three percent of our patients achieved complete resolution of their GERD symptoms and significant improvement of EE ^32^.

Recently, Holmberg et al. ^28^, in Sweden, including many patients submitted to LGB, have contested this long-standing notion (with an extended follow-up, mean of 4.9 years). Interestingly, they found that GERD (defined as postoperative reflux as residual or recurring symptoms with the use of acid suppression medications beyond 6 months postoperatively) persisted in 48.8% of patients within 2 years after LGB and continued for up to 10 years after surgery. Despite these findings, the authors concede that LGB remains the most effective bariatric procedure in reducing GERD for the reasons mentioned earlier ^3^ ^,^ ^28^.

As it is possible to observe, the appearance of reflux symptoms after LGB has been reported to be as high as 22% of patients (range, 3.8-22%). The reported main symptoms are pain (27.3-44.7%), dysphagia (12.5%), respiratory symptoms (17%), and vomiting (12.5%). EE and Barrett´s esophagus have been observed in a wide range of percentage (2.4-10.6% and 0-17.6%, respectively) ^5^ ^,^ ^7^ ^,^ ^13^ ^,^ ^20^ ^,^ ^23^ ^,^ ^27^ ^,^ ^28^ ^,^ ^29^ ^,^ ^31^ ^,^ ^33^ ^,^ ^34^ ^,^ ^39^ ^,^ ^40^ ^,^ ^41^ ^,^ ^44^.

The enormous discrepancy and variability observed in the reported data can be explained because it is difficult to provide an exact percentage, due to many of these patients have been initially operated on by other centers and, subsequently, derivate to other units for evaluation and treatment.

Few studies have been dedicated to determining the causes of GERD after LGB. These studies did not refer to the anatomical aspects and pathophysiological mechanisms involved in both surgical aspects. There is no specific mention in the literature regarding the existence or repair of a concomitant HH during the first surgery or the size of the gastric pouch, an important detail that many surgeons miss during the first surgery.

Several explanations for developing gastroesophageal symptoms after LGB have been proposed (Table 5).

**Table 5 -:** Proposed pathophysiologic and anatomic mechanisms for the genesis of gastroesophageal reflux after laparoscopic gastric bypass.

1. Depending on gastric anatomic fators	Hiatal hernia Cardia dilatation Acid pouch Large gastric pouch Asymmetric pouch (Candy cane pouch) Anastomotic stricture with gastric retention Gastrogastric fistula
2. Depending on the esophageal funcion	Incompetent LES or increased transient relaxation Esophageal motor dysfunction (hypomotility) Others: ectopic gastric mucosa, eosinophilic esophagitis, esophageal hypersensitivity
3. Depending on jejunal limb	Length Efferent loop síndrome Roux-en-Y syndrome with stasis Mechanical obstruction
4. Others	Zollinger-Ellison syndrome, psychiatric diseases

The gastroesophageal symptoms are mainly associated with the anatomic and functional mechanisms. In this article, we analyze the most common findings that are responsible for the appearance of these symptoms:

### 1. Anatomic defects

Hiatal hernia: It is one of the most frequent etiology symptoms^5^ ^,^ ^38^ ^,^ ^42^ ^,^ ^43^, as has also been described by many authors. It is important to emphasize that even in patients without symptoms, during the first surgery, it is essential to rule out the presence of a small HH so that the status of the hiatus can be reviewed during the procedure of closure of the hiatus, with fixation of the pouch below the hiatus, and avoid HH in the postoperative period. However, most surgeons omit this maneuver during the performance of LFB. HH must be identified and repaired during surgery to avoid later symptoms ^10^ ^,^ ^11^. Surgical errors committed during performing LGB could lead to the appearance of the complications described, which must be avoided.

HH occurrence after LGB can occur quite frequently after gastric bypass ^5^. The relatively small size of the gastric pouch, dissection injury to sling fibers, and tissue strength changes related to rapid weight loss may all predispose patients to postoperative HH occurrence after gastric bypass. In the event of acutely worsening postprandial epigastric pain, nausea, dysphagia, and/or vomiting, the presence of HH must be suspected. Diagnostic imaging and upper endoscopy may reveal the diagnosis of gastric bypass pouch herniation into the esophageal hiatus ^14^ ^,^ ^37^. Signorini et al. reported 10% of HH after LGB is responsible for symptoms and EE ^39^.

Large proximal gastric and acid pouch: Abnormal acid reflux is not expected after LGB, given the small size of the gastric pouch and reduced gastric capacity ^5^ ^,^ ^23^ ^,^ ^35^ ^,^ ^36^ ^,^ ^38^ ^,^ ^39^. A decrease in acid secretion secondary to the creation of a small pouch with a very low amount of parietal cell mass and Roux-en-Y gastrojejunostomy with a long alimentary loop avoid bile reflux completely, improving symptoms and healing of EE. A deficient reduction of acid secretion is probably due to a large gastric pouch. Boberly et al. reported that 10% of the patients had an enlarged gastric pouch; however, 61% of the patients had abnormal esophageal acid exposure ^5^. We also observed patients with erosive jejunitis, probably in the context of acid hypersecretion.

Anastomotic stricture or distal obstruction could cause gastric retention and enlargement of the gastric pouch and GERD. Dysphagia can be associated with stomal stenosis after LGB, its prevalence ranging from 3.1% to 10.4% ^14^, confirmed during upper gastrointestinal endoscopy. Boberly et al. reported stomal stenosis in 10.4% of his cases associated with bleeding (2.8%) and food impaction (0.8%) ^5^.

Marginal ulcer or jejunitis incidence has been described as ranging from 0.1% to 8.6% after LGB. It is most prevalent in the first year after surgery, but not restricted to the first year, with a mean time between surgery and the first symptoms of approximately 4 months ^34^ ^,^ ^35^. Some of these patients might refer to GERD symptoms; however, it is uncommon to find clear esophagitis in LGB patients complaining of upper gastrointestinal symptoms ^9^ ^,^ ^44^.

Candy cane configuration was the cause for symptoms in one case of our experience.

### 2. Abnormal function of LES and esophageal motility

Incompetent LES was described many years ago after distal gastrectomy and confirmed more recently by other authors, ranging from 15.7% to 62% of the cases after LGB ^10^ ^,^ ^11^. Most of these cases were associated with the presence of HH ^5^ ^,^ ^42^. The cause of sphincter incompetence is the section of the sling fibers, and modification of His´s angle and cardia dilatation, which are the most important determinants of the sphincter strength ^9^ ^,^ ^10^ ^,^ ^18^.

Motor disorders as another cause of postoperative dysphagia have been mentioned in the literature, specially hypotonicity of esophageal waves that occur in 5-19.9% of the patients ^2^ ^,^ ^4^ ^,^ ^13^ ^,^ ^21^ ^,^ ^18^. This finding was also observed in 17% of the patients in our study. Valezi ^43^ reported abnormal manometry findings in 62.9% of the patients, 53% had changes in amplitude contraction, and 19.6% had abnormal peristalsis 1 year after surgery. In the present study, we identified patients with hypomotility. However, dysphagia occurs mainly due to anatomical rather than physiological factors.

Impedance measurement can provide more appropriate information about the content of refluxes. Unfortunately, this was not routinely available to us during the study.

### 3. Jejunal limb factors

Distal obstruction: Small bowel obstruction is rare with a reported incidence of 5%, and it is secondary to other mechanisms such as internal hernia or transmesenteric obstruction. However, symptoms secondary to these complications are quite different, and the predominant symptoms are pain and vomiting.

The symptoms of gastroesophageal reflux observed after bypass should theoretically not be related to the length of the Roux limb because, in these patients, the length of the loop is, at least, 150 cm.

### 4. Other factors and Zollinger-Ellison syndrome

Hypergastrinemia does not seem to cause increased acid secretion since normal serum gastrin levels have been found after gastric bypass; however, very limited information is available^21^. In fact, in our study, only one patient showed hypergastrinemia, which could be related to a retained antrum.

The increased secretion of acid after LGB most likely occurs due to an enlarged gastric pouch containing an increased number of parietal cells, associated with anatomic anomalies causing gastric retention. We also observed patients with erosive jejunitis, probably in the context of acid hypersecretion.

In cases with a negative pH test, the symptoms were more probably due to ingestion and retention in the herniated gastric pouch or the saccular formation of the blind loop associated with foods with high acid content (tomato sauce, spices sauce, or other similar) that can promote mucosal damage.

Table 6 shows a summary of the reported data concerning this topic.

**Table 6 -:** Gastroesophageal symptoms, endoscopy, radiology, and esophageal functional studies after laparoscopic gastric bypass.

	% of patients mean (range)	Authors reporting data	References
Symptoms	13.9% (8-22)	10	1,5,11,27,30, 32,34,35,38,39
Endoscopy	29.6% (5.1-57.1)	10	1,5,11,20-23,38,32,33
Erosive esophagitis	6.6% (0-17.6)	5	2,5,19,22,39
Barrett´s esophagus	10.4% (0-24)	3	5,22,39
Hiatal hernia	6.5% (0.1-8.6)	4	5,19,33,34
Marginal ulcer/jejunitis	7.8% (3.1-10.4)	2	5,13
Anastomotic stricture			
Radiology			
Hiatal hernia	36.6% (20-53.2)	3	5,7,19
Enlarged pouch	7.5% (4.5-10.6)	8	5,7,19,22,34, 35,37,38
Pouch gastric fistula	4.3%	1	5
Manometry*			
Hypotensive LES	57.8%	1	5
Motility disorders	36.6% (14.1-58)	4	5, 20, 35,38
1 author reported % of patients presenting hypotensive LES			
Four authors reported functional findings:			
LESP (mmHg) = 11.2±4.5 (range 8.1-22.0)			
LES Length (cm) =1.9±1.6 (range 1.2-5)			
24-h pH monitoring	29.6% (12.5-61.4)	4	5,11,22,38
Pathologic reflux			

Finally, regarding the treatment, it will choose based on the findings after a complete evaluation, local resources, and skills, and if necessary, refer the patient to a specialist center. The strength of this study is that it is one of the few objective investigations focused on determining the possible pathophysiologic and anatomical factors for the gastroesophageal symptoms after LGB and this was a prospective study. However, the limitations are that a small number of patients were included and it is not possible to have a matched control group.

## CONCLUSION

The correct execution of the surgical technique during the initial procedure, such as leaving a small gastric pouch, identification and repair of hiatal hernia, avoid redundant or very long blind loop, avoid anastomotic stricture, torsion or compression of the efferent alimentary loop, can avoid reflux complaints after surgery.

**Disclosure statement:** The authors declare that they do not have any material interest related to the research described in this article (nothing to disclose).

All procedures involving human participants were in accordance with the Ethic Institutional Committee and with the revised Helsinki Declaration (Brazil 2013) and its later amendments or comparable ethical standards. No patient data appear in this article. Written informed consent was obtained from all participants included in the study.
